# Palliative Efficacy of High-Dose Stereotactic Body Radiotherapy Versus Conventional Radiotherapy for Painful Non-Spine Bone Metastases: A Propensity Score-Matched Analysis

**DOI:** 10.3390/cancers14164014

**Published:** 2022-08-19

**Authors:** Kei Ito, Kentaro Taguchi, Yujiro Nakajima, Hiroaki Ogawa, Keiko Nemoto Murofushi

**Affiliations:** 1 Division of Radiation Oncology, Department of Radiology, Tokyo Metropolitan Cancer and Infectious Diseases Center, Komagome Hospital, 3-18-22 Honkomagome, Bunkyo-ku, Tokyo 113-8677, Japan; 2 Department of Radiological Sciences, Komazawa University, 1-23-1 Komazawa, Setagaya-ku, Tokyo 154-8525, Japan; 3 Department of Radiation Oncology, Tohoku University Graduate School of Medicine, 1-1 Seiryo-machi, Aoba-ku, Sendai 980-8574, Japan

**Keywords:** non-spine bone metastases, radiotherapy, stereotactic body radiotherapy, pain response, propensity score-matched analysis

## Abstract

**Simple Summary:**

As the superiority of stereotactic body radiotherapy (SBRT) over conventional external beam radiotherapy (cEBRT) for painful non-spine bone metastases remains controversial, we conducted propensity score-matched analysis. Our results showed that the 3-month pain response rate after SBRT was significantly higher than that after cEBRT. Therefore, these findings suggest that large-scale randomized controlled trials are warranted to compare SBRT with cEBRT for painful bone metastases not involving the spine.

**Abstract:**

(1) Background: The superiority of stereotactic body radiotherapy (SBRT) over conventional external beam radiotherapy (cEBRT) in terms of pain palliation for bone metastases remains controversial. (2) Methods: This propensity score-matched study compared the overall pain response (OR) 3 months after radiotherapy among patients with painful (≥2 points on a 0-to-10 scale) non-spine bone metastases. Patients with lesions that were treated with SBRT or cEBRT and whose pain scores were evaluated 3 months after radiotherapy were included in this study. Pain response was evaluated according to the International Consensus Criteria. (3) Results: A total of 234 lesions (SBRT, *n* = 129; cEBRT, *n* = 105) were identified in our institutional database. To reduce the confounding effects, 162 patients were selected using a propensity score-matched analysis (*n* = 81 for each treatment). The OR rate at 3 months after SBRT was significantly higher than that after cEBRT (76.5% vs. 56.8%; *p* = 0.012). A noteworthy finding of our study is that the same trend was observed even after 6 months (75.9% vs. 50.0%; *p* = 0.011). The 1-year local failure rates after SBRT and cEBRT were 10.2% and 33.3% (*p* < 0.001), respectively. (4) Conclusions: Our findings suggest that SBRT is superior to cEBRT for pain palliation in patients with non-spine bone metastases.

## 1. Introduction

Bone is a common site of metastasis in advanced cancer [1,2,3], and bone metastases often result in debilitating cancer-related pain [3]. Conventional external beam radiotherapy (cEBRT) is the standard of care for painful bone metastases [4]. Its palliative efficacy and few associated adverse effects (AEs) have been demonstrated in multiple phase III trials and meta-analyses, regardless of the dose fraction schedules, such as 8 Gy in a single fraction, 20 Gy in five fractions, and 30 Gy in 10 fractions [4,5].

However, cEBRT also has several limitations. First, cEBRT may not always be effective. Previous studies have shown that the overall pain response (OR) rate and complete pain response (CR) rate were approximately 60% and 25%, respectively [5]. Second, the pain response duration was short (median, 20–24 weeks) [6]. Third, it is less effective against radioresistant tumors than against more radiosensitive primary tumors [7]. As advances in systemic therapy extend the life expectancy of patients with metastatic diseases, these limitations are becoming a matter of great concern.

Stereotactic body radiotherapy (SBRT) is a high-precision radiotherapy technique that delivers a high ablative biological dose in one to five high-dose fractions, while sparing adjacent organs. SBRT has emerged as an attractive alternative to cEBRT for spinal metastases [8] and has also been used in clinical practice in patients with bone metastases not involving the spine [9]. SBRT can theoretically overcome the limitations of cEBRT; however, few studies have reported its efficacy for pain relief in bone metastases not involving the spine [10,11]. Therefore, we conducted a propensity score-matched (PSM) analysis to clarify whether SBRT is superior to cEBRT in terms of pain relief in non-spine bone metastases.

## 2. Materials and Methods

### 2.1. Patients

The database of Tokyo Metropolitan Komagome Hospital was retrospectively reviewed to identify patients treated with radiotherapy for pain palliation in non-spine bone metastases. Patients were included in this study if they met the following criteria: (i) presence of painful bone metastases not involving the spine (numerical rating score [NRS] ≥2 points); (ii) painful osseous lesions treated with SBRT from February 2013 to January 2022 or with cEBRT from February 2013 to May 2018; and (iii) evaluation of the index pain 3 months after radiotherapy. “Non-spine bone” was defined as all bones excluding the cervical spine, thoracic spine, lumbar spine, sacral spine, and coccyx. Because we performed SBRT for all patients with painful bone metastases not involving the spine who were in a good general condition since June 2018 based on the excellent outcomes of our clinical trial [11], the irradiation period of cEBRT was limited to before June 2018 to minimize patient selection bias. The following patients were excluded from this study: (i) patients with target bones in the base of the skull; (ii) patients with radiation-sensitive tumors, including malignant lymphoma, myeloma, and germ cell tumors; and (iii) patients with other painful lesions that were irradiated simultaneously or within 3 months after non-spine bone radiotherapy, because the presence of other lesions with severe pain makes it difficult to assess the pain of interest.

This retrospective study protocol was approved by Komagome Hospital’s Institutional Ethics Review Board (approval number: 2906). Informed consent was obtained in the form of an opt-out option on the website.

### 2.2. Stereotactic Body Radiotherapy

**Figure 1:**
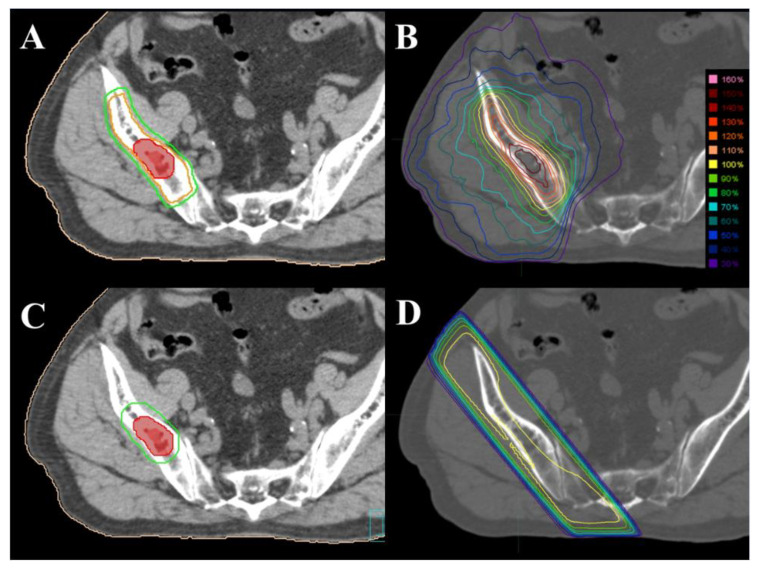
Axial computed tomography images with contours (red = gross tumor volume, orange = clinical target volume, and green = planning target volume) and dose distribution of stereotactic body radiotherapy (**A**,**B**) and conventional external beam radiotherapy (**C**,**D**) for coxal bone metastases.

The SBRT technique, discussed in detail in a previous publication [11], is briefly summarized here. The planning CT simulation was performed with a slice thickness of 2 mm. The gross tumor volume (GTV) was set based on the simulation CT with reference to the diagnostic enhanced CT, magnetic resonance imaging (MRI), and positron emission tomography images. Next, 5−10 mm of additional GTV was used to expand the clinical target volume (CTV), while considering the anatomical barrier. In addition to the above CTV, the CTV margin was expanded by 20–30 mm within the targeted bone [12] owing to anatomical continuity. A 3 mm margin was added to the CTV to calculate the planning target volume (PTV) (Figure 1A).

The prescribed dose (PD) was 30 or 35 Gy in five fractions; however, some lesions that were irradiated with 24 Gy in two fractions along with spinal metastases were included in the study. The planning goals were as follows: 95% of the PTV should receive 100% (allowed down to 85%) of the PD (85% PD ≤ PTV D_95%_ ≤ 100 % PD), and the maximum dose should be ≤170% of the PD (PTV D_max_ ≤ 170% PD). The treatment was delivered by TrueBeam (Varian Medical Systems, Palo Alto, CA, USA) and Vero4DRT (Mitsubishi Heavy Industry, Tokyo, Japan) using intensity-modulated radiotherapy techniques (Figure 1B). The dose calculation and optimization for the SBRT plans were performed using Eclipse (Varian Medical Systems), RayStation (RaySearch Laboratories, Stockholm, Sweden), and iPlan (BrainLab AG, Feldkirchen, Germany).

### 2.3. Conventional External Beam Radiotherapy

The planning CT simulation was performed with a slice thickness of 3 mm. The GTV was set in the same manner as for SBRT. An additional GTV of 0−5 mm was used for expansion to the CTV, while considering the anatomical barrier. A 5 mm margin was added to the CTV to calculate the PTV (Figure 1C). The PD of cEBRT was 8 Gy in a single fraction, 20 Gy in five fractions, or 30 Gy in 10 fractions according to the recommendation of the American Society for Radiation Oncology [4]. Radiotherapy was administered with a three-dimensional conformal technique using 2−4 ports, and the planning goal was to cover the PTV with approximately 90–110% of the PD (Figure 1D). Intensity-modulated radiotherapy or volumetric-modulated arc therapy were not used.

### 2.4. Endpoints

The primary endpoint of the present study was the OR rate for pain from non-spine bone metastases 3 months after radiotherapy. The secondary endpoints comprised OR rates at 1 and 6 months; CR rates at 1, 3, and 6 months; local failure (LF) rate; overall survival (OS); and AEs.

### 2.5. Evaluation

Pain status at the treated index lesion was measured on an NRS of 0–10, noting the worst score in the previous 3 days. Pain response was judged according to the criteria defined by the International Consensus Pain Response Endpoints and evaluated according to the NRS and the amount of analgesic consumed [13]. Extrapolating from the Spine Response Assessment in Neuro-Oncology group recommendation [14], LF was defined as tumor progression based on MRI or CT and was calculated in months (from the starting date of radiotherapy to the date of tumor progression or the date of the last follow-up for the imaging study). OS was defined as the interval between radiotherapy and the most recent follow-up or death due to any cause. AEs were evaluated according to the Common Terminology Criteria for Adverse Events version 5 of the National Cancer Institute [15]. Acute AEs and late AEs are defined as AEs arising within 90 days and after 90 days of the start of the protocol treatment, respectively.

### 2.6. PSM Analysis

The propensity scores were generated using multivariable logistic regression models predictive of the radiation technique (SBRT/cEBRT), based on patient characteristics (age and Eastern Cooperative Oncology Group performance status [ECOG PS]), and tumor characteristics (radiation sensitivity [16,17,18], the presence or absence of a radiation history [17,19], and the degree of pain). The one-to-one match started with an exact match of radiation sensitivity, followed by a nearest-neighbor match. Standardized differences for the covariates were calculated to assess the comparability of the matched cohorts and matched to be less than 0.1 for all covariates. The matching procedure was implemented using the “Matching” package in R [20].

### 2.7. Statistical Analysis

The balance of the baseline characteristics between the two groups was assessed by Fisher’s exact/ Chi-squared test (categorical variables) or the Mann–Whitney U test (continuous variables). Fisher’s exact test was used to compare the pain response (OR and CR) rates between the two groups. As patient death without tumor recurrence was regarded as a competing risk factor, LF was estimated using the cumulative incidence function adjusted for the competing risk of death and compared using Gray’s test for equality. OS was estimated using the Kaplan–Meier method, and the log-rank test was used to compare OS between groups. A *p* value of <0.05 was considered statistically significant. All statistical analyses except for matching were performed using the EZR software, version 1.54 [21].

## 3. Results

### 3.1. Baseline Patient Characteristics

**Figure 2:**
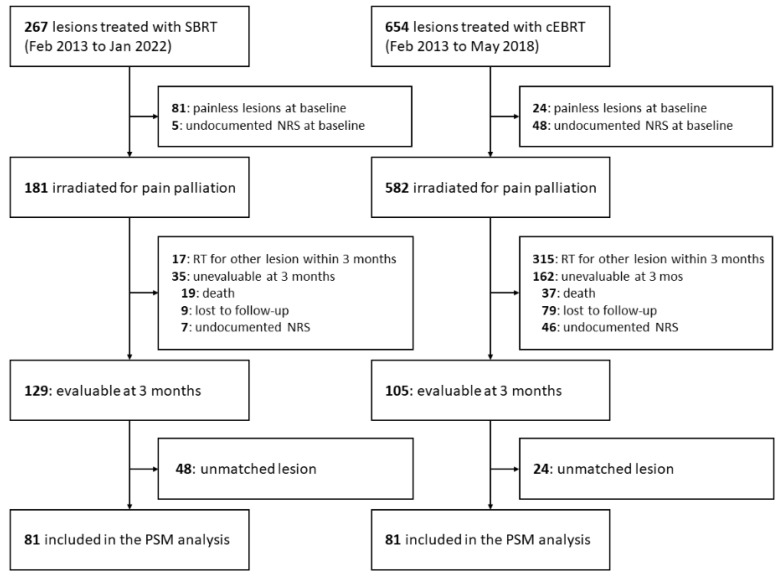
Study profile.

The study profile is shown in Figure 2.

During the study period, 921 non-spine bone lesions underwent SBRT (*n* = 267) or cEBRT (*n* = 654). Eighty-six and seventy-two lesions were excluded at the baseline in the SBRT and cEBRT groups, respectively, and 52 and 477 lesions were excluded at 3 months after radiotherapy in the SBRT and cEBRT groups, respectively. A total of 234 lesions (SBRT, *n* = 129; cEBRT, *n* = 105) met the inclusion criteria.

Patient characteristics of the whole cohort are summarized in Table 1. The SBRT group included a larger number of patients in good general condition (PS 0: 43.4% vs. 19.0%), radioresistant primary tumors (35.7% vs. 24.8%), and fewer limb lesions (8.5% vs. 31.4%) than the cEBRT group.

The pain responses in each group are summarized in Table 2. A significant difference was observed in OR rate at three months between SBRT and cEBRT groups (77.5% vs. 54.3%; *p* < 0.001). The OR rate at 1 and 6 months after SBRT was significantly higher than that after cEBRT (76.9% vs. 58.7%; *p* < 0.01 and 72.2% vs. 48.0%; *p* < 0.01, respectively).

### 3.2. Patient Characteristics in the PSM Cohort

The PSM analysis for reduction of confounding effects identified 81 matched pairs of lesions in each group. The matched groups of patients had similar baseline characteristics (Table 2), including age, PS, radiation sensitivity, the presence or absence of radiation history, and degree of pain (PS, *p* = 0.39; others, *p* ≥ 0.83).

### 3.3. Treatment Efficacy in the PSM Cohort

**Figure 3:**
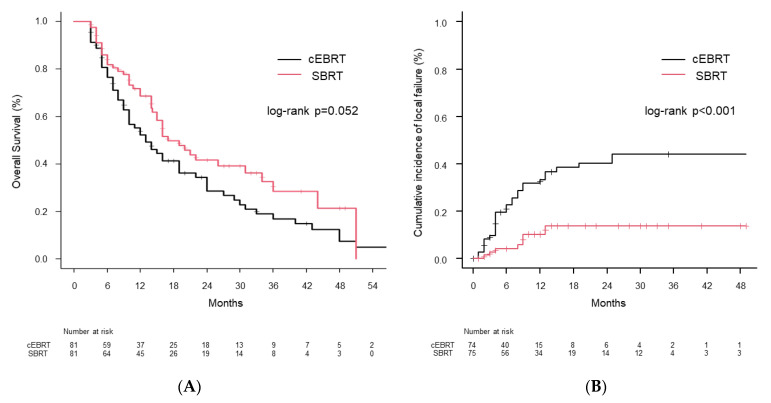
(**A**) Kaplan−Meier curves of overall survival and (**B**) cumulative incidence of local failure after radiotherapy in the propensity score-matched cohort.

The median follow-up duration after radiotherapy was 12 months (SBRT group: 14 [range, 3–51] months, cEBRT group: 10 [range, 3–66] months). The matched SBRT and cEBRT groups had similar OS rates at 6 months (82.0% vs. 76.7%) and 1 year after radiotherapy (68.6% vs. 52.3%, *p* = 0.052; Figure 3A).

Pain response at 1 and 6 months after radiotherapy was evaluated in 75 and 58 lesions, respectively, in the SBRT group and in 73 and 42 lesions, respectively, in the cEBRT group.

Significant difference was observed in the OR rate at 3 months after radiotherapy between the SBRT and cEBRT groups (76.5% vs. 56.8%; *p* = 0.012; Table 2) in the PSM cohort as well. The OR rates at 1 and 6 months after SBRT were significantly higher than those for cEBRT (77.3% vs. 61.6%, *p* = 0.049 and 75.9% vs. 50.0%, *p* = 0.011, respectively). Regarding the CR rate, the difference was not confirmed at 1 month after radiotherapy (34.7% vs. 31.5%; *p* = 0.729). However, the CR rate of SBRT increased thereafter to more than 50%, and this analysis showed a significant difference, favoring SBRT to cEBRT at 3 months (51.9% vs. 34.6%; *p* = 0.039) and 6 months (56.9% vs. 35.7%; *p* = 0.044) after radiotherapy.

The 1-year imaging local failure rates after SBRT and cEBRT were 10.2% and 33.3% (*p* < 0.001), respectively (Figure 3B).

### 3.4. AEs in the PSM Cohort

The AEs in the PSM cohort are shown in Table 3. Grade 3 or more acute AEs were confirmed in none of the SBRT groups and three of the cEBRT groups (3.7%). cEBRT did not cause grade 2 or higher late AEs, whereas seven patients treated with SBRT (8.6%) experienced late AEs. The seven (five painless and two painful) fractures caused by SBRT (8.6%) comprised four coxals, two ribs, and one clavicle. The median time to fracture was 4 months (range 2–27 months). All lesions with fractures had negative histories of radiotherapy before SBRT.

## 4. Discussion

The present PSM study showed that SBRT had better palliative efficacy for painful bone metastases not involving the spine than cEBRT at 3 months after radiotherapy. In addition, some advantages of SBRT, such as a high CR rate and long-term (e.g., 6 months) pain control, were observed, which could be due to the excellent local control of SBRT.

Although the present study demonstrated the superiority of SBRT for pain palliation, randomized controlled trials comparing the pain-relieving effects between SBRT and cEBRT for bone metastases reported paradoxical results: superiority of SBRT over cEBRT [10,22] and non-superiority [23,24,25,26,27]. Consequently, the superior effectiveness of SBRT is still uncertain. Additionally, although the reasons for the discrepancy in results are unclear, we postulate that the following factors may have contributed to the difference: (i) difference of prescribed SBRT dose; (ii) spine or non-spine bone; (iii) unbalanced allocation to each group (e.g., PS [25], radiation sensitivity); and (iv) intent-to-treat or per-protocol analyses (owing to high death rate and loss of follow-up).

In SBRT for bone metastases, the irradiation method differs depending on the presence or absence of the spinal cord within the irradiation fields; hence, treatment methodologies for spinal and non-spine bone metastases were developed separately [28]. Although pain relief is one of the most important therapeutic aims of SBRT [28], few others have reported pain palliation in non-spine bone SBRT [10,11]. A randomized phase II trial reported by Nguyen et al. indicated that SBRT had more pain responders than cEBRT (per protocol set at 3 months: 72% vs. 49%; *p* = 0.03) [10]. Another single-arm phase II trial showed a 3-month OR rate of 78% in the per protocol analysis [11]. The present study showed that the 3-month OR rates for SBRT and cEBRT were 77% and 57%, respectively. These three studies suggest that SBRT has better palliative efficacy than cEBRT for non-spine bone metastases and warrant large-scale randomized controlled trials to compare SBRT with cEBRT in this clinical setting. 

Assuming that SBRT is superior to cEBRT, we considered the contributing factors in terms of the dose and target size. Regarding radiation dose, some meta-analyses showed a positive correlation between the prescribed dose and local control rate in spinal SBRT [29,30]. In contrast, high-dose radiation can cause painful fractures [29] and inflammation in the surrounding tissues [14]. Therefore, SBRT for pain relief requires an exquisite balance in the treatment intensity. In the above randomized phase II trial on SBRT for painful non-spine bone metastases [10], a single fraction SBRT of 16 Gy (biologically effective dose [BED] of 41.6 Gy with α/β assumed to be 10 for tumors) showed a higher OR rate and longer pain control duration than an SBRT of 12 Gy (BED of 26.4 Gy). The present study and our previous trial [11] using a higher dose (BED of 48 or 59.5 Gy) achieved comparable pain relief to that of the randomized phase II trial. These findings suggest that high-dose SBRT with a BED of 40–60 is suitable for patients with painful non-spine bone metastases.

With regards to spinal metastases, while the field of cEBRT includes spinal lesions plus one segment above and below, SBRT includes only the involved spine as the target. Although the merits and demerits of different field sizes are unknown for pain relief, there are some reports showing that a large field size may induce pain relief [31,32]. In a randomized phase II trial of non-spine bone SBRT [10], a small CTV margin of 2–3 mm was used (the protocol described that the PTV was defined as the GTV plus a 5-mm circumferential margin). Our group adopted a large CTV margin of 20–30 mm [11,12]. However, since these response rates were not significantly different, the effect of the SBRT’s target size on pain relief may be limited.

In the present study, the CTV margin of SBRT was larger than that of cEBRT. The large CTV margin (20–30 mm) in SBRT was determined based on a previous analysis regarding patterns of intraosseous recurrence [12]. Out-of-field recurrences in the same bone were observed in more than 40% of cases treated via SBRT with a CTV margin of 5–10 mm, and the mean distance to the recurrent tumor from the initial bone metastasis was 34 mm (range, 15–55 mm) [12]. On the contrary, the irradiated bone volume of cEBRT was often larger than that of SBRT, owing to the poor dose concentration of cEBRT (like the case in Figure 1). Therefore, it was difficult to determine the correlation between pain relief effect and target size in this study.

Ablative irradiation with SBRT has high antitumor effects, resulting in a high tumor control rate [8,33]. If this assumption holds, SBRT is expected to improve the CR rate [8,22] and long-term pain control [33]. In the present study, the 1-month CR rate was only 30%, which is comparable to that of cEBRT. However, the CR rate increased to more than 50% in 3 months and was maintained up to 6 months after SBRT. These findings may indicate the timing of occurrence of the maximum effect of SBRT.

We performed bone SBRT for patients in a good general condition. Contrastingly, cEBRT was provided to patients with all statuses of general condition. Hence, we selected only those patients with a good general condition among the cEBRT cohort and matched them to the SBRT group. Therefore, the finding of no significant difference in OS among the two groups may be attributed to PSM. However, OS in the SBRT group was comparatively longer than that in the cEBRT group. The high OR rate and low local failure rate of SBRT may have contributed to the maintenance of the general condition and activities and subsequent prolongation of survival (alternatively, the longer OS may have been owing to a selection bias or advances in systemic therapy).

Fractures are relatively common AEs of bone SBRT. High-dose radiation damages the bone matrix and degrades the vascular supply to the bone, leading to bone fractures [34]. The incidence rate of vertebral compression fractures within 5 years post-SBRT was significantly higher than that post-cEBRT (22% vs. 7%, *p* = 0.044) according to a matched pair analysis [35]. The present study confirmed more frequent fractures of SBRT compared to cEBRT (8.6% vs. 0%). However, the fractures were a painless AE in more than 70% lesions. In the SC.24 trial that prospectively evaluated the palliative effects of spine SBRT and cEBRT, 94% (30 of 32) of vertebral compression fractures were painless [22]. According to the above findings, SBRT often causes fractures, but the impact of the fractures on quality of life is limited.

The current study has some limitations. First, this was a retrospective cohort study and not a randomized controlled trial, although PSM analysis was used to minimize selection bias. Second, the sample size was insufficient to obtain any conclusive results. Third, patients with osseous oligometastases were included in the SBRT group only. A total of 33 lesions (41%) in the SBRT group were oligometastases (24 oligometastases and 9 oligo-progressive diseases), and the SBRT was performed for curative intent. Contrarily, all cEBRTs were conducted for palliative intent. It cannot be denied that the difference might have affected the pain response because of the presence of other painful lesions.

## 5. Conclusions

The present PMS analysis showed that SBRT for non-spine bone metastases was superior to cEBRT in terms of the 3-month pain response rate and demonstrated some advantages, such as a high CR rate and long-term pain control. The findings of this study warrant a large-scale randomized controlled trial comparing SBRT with cEBRT in a clinical setting.

## Figures and Tables

**Table 1:** Patient and tumor characteristics of all lesions and matched lesions.

Characteristic	All Lesions			Matched Lesions		
	SBRT Group (*n* = 129)	cEBRT Group (*n* = 105)	*p*-Value	SBRT Group (*n* = 89)	cEBRT Group (*n* = 81)	*p*-Value
Sex Male/Female	84/45	65/40	0.68	51/30	55/26	0.62
Age, years Median (range)	68 (28–85)	67 (29–90)	0.63	68 (28–85)	67 (46–90)	0.83
ECOG performance status 0/1/2/3/4	56/60/9/3/1	20/50/20/12/3	<0.001	25/44/8/3/1	20/47/9/4/1	0.39
Primary malignancy Radioresistant tumor Colorectal Renal cell Sarcoma/osteosarcoma Thyroid Hepatocellular Melanoma Radiosensitive tumor Prostate Breast Other Lung Bladder Esophagus Uterus Head and Neck Others	46 (35.7%) 11 22 7 3 1 2 22 (17.1) 15 7 61 (47.3) 34 2 0 9 5 11	26 (24.8%) 10 8 3 3 2 0 17 (16.2) 9 8 62 (59.0) 48 3 3 1 1 6	0.15	22 (27.2%) 4 11 6 0 1 0 16 (19.8) 10 6 43 (53.1) 27 1 0 5 2 8	23 (28.4%) 8 7 3 3 2 0 16 (19.8) 8 8 42 (51.9) 32 3 3 0 0 4	1.00
Site treated * Coxal Rib Sternum Clavicle Scapula Limb (humerus/femur) Skull	70 19 11 3 11 11 (4/7) 7	43 18 1 2 12 33 (5/28) 0	<0.001	43 15 6 3 6 5 (3/2) 6	30 16 2 2 10 24 (5/19) 0	<0.001
Bone lesion Lytic/blastic/mixed	68/19/42	53/32/20	<0.01	40/11/30	44/20/17	0.047
Pain score 2–4 (mild) 5–7 (moderate) 8–10 (severe)	37 (28.7%) 52 (40.3) 40 (31.0)	32 (30.5%) 41 (39.0) 32 (30.5)	0.96	22 (27.2%) 35 (43.2) 24 (29.6)	25 (30.9%) 33 (40.7) 23 (28.4)	0.92
Radiation history +/−	31/98	21/84	0.53	18/63	16/65	0.85
Radiation dose 8 Gy in 1 fx 20 Gy in 5 fx 30 Gy in 10 fx 24 Gy in 2 fx (with spine) 30 Gy in 5 fx 35 Gy in 5 fx	0 0 0 7 22 100	29 20 56 0 0 0	NA	0 0 0 2 15 64	22 19 40 0 0 0	NA

cEBRT, conventional external beam radiotherapy; ECOG, Eastern Cooperative Oncology Group; NA, not applicable; SBRT, stereotactic body radiotherapy. * Several targets included lesions across the sites in each group.

**Table 2:** Pain response over time.

		All Lesions, Number (%)			Matched Lesions, Number (%)		
		SBRT Group	cEBRT Group	*p*-Value	SBRT Group	cEBRT Group	*p*-Value
1 month	Responders CR + PR	93/121 (76.9) 41 + 52	54/92 (58.7) 27 + 27	<0.01	58/75 (77.3) 26 + 32	45/73 (61.6) 23 + 22	0.049
	Non-responders PP + IR	28/121 (23.1) 5 + 23	38/92 (41.3) 4 + 34		17/75 (22.7) 2 + 15	28/73 (38.4) 2 + 26	
3 months	Responders CR + PR	100/129 (77.5) 66 + 34	57/105 (54.3) 35 + 22	<0.001	62/81 (76.5) 42 + 20	46/81 (56.8) 28 + 18	0.012
	Non-responders PP + IR	29/129 (22.5) 10 + 19	48/105 (45.7) 9 + 39		19/81 (23.5) 4 + 15	35/81 (43.2) 8 + 27	
6 months	Responders CR + PR	70/98 (72.2) 53 + 17	24/50 (48.0) 17 + 7	<0.01	44/58 (75.9) 33 + 11	21/42 (50.0) 15 + 6	0.011
	Non-responders PP + IR	27/98 (27.8) 13 + 14	26/50 (52.0) 5 + 21		14/58 (24.1) 4 + 10	21/42 (50.0) 5 + 16	

cEBRT, conventional external beam radiotherapy; CR, complete response; IR, indeterminate response; PP, pain progression; PR, partial response; SBRT, stereotactic body radiotherapy.

**Table 3:** Adverse effects of radiotherapy in the PSM cohort.

	SBRT Group (*n* = 81)			cEBRT Group (*n* = 81)		
	Grade 2	Grade 3	Grade 4–5	Grade 2	Grade 3	Grade 4–5
Acute phase Pain Nausea Dermatitis Oral mucositis Enterocolitis Fracture Total	1 2 3 1 1 1 9 (11.1%)	0 0 0 0 0 0 0	0 0 0 0 0 0 0	0 2 0 0 0 0 2 (2.5%)	0 1 0 0 1 1 3 (3.7%)	0 0 0 0 0 0 0
Late phase Fracture Dermatitis Pneumonitis Hematuria Limb edema Total	1 1 1 0 2 5 (6.2%)	0 0 1 1 0 2 (2.5%)	0 0 0 0 0 0	0 0 0 0 0 0	0 0 0 0 0 0	0 0 0 0 0 0

cEBRT: conventional external beam radiotherapy; PSM: propensity score-matched; SBRT: stereotactic body radiotherapy.

## Data Availability

The datasets used and/or analyzed during this study are available from the corresponding author on reasonable request.
